# GABA Mediates the Enhancement of Maize (*Zea mays* L.) Saline–Alkali Tolerance Through DJ Bacterium

**DOI:** 10.3390/microorganisms14061271

**Published:** 2026-06-05

**Authors:** Jianing Zhao, Hanna Wang, Yajun Fan

**Affiliations:** College of Life Sciences, Changchun Normal University, Changchun 130032, China; 15024815975@163.com (J.Z.); wanghn15504427066@126.com (H.W.)

**Keywords:** strain DJ, GABA, saline–alkali stress, maize

## Abstract

Soil salinization is considered a major abiotic stress limiting the sustainable development of agriculture globally. Utilizing plant growth-promoting rhizobacteria (PGPR) to enhance crop tolerance under saline–alkali stress constitutes a sustainable and promising strategy. This study focuses on *Enterobacter cloacae* strain DJ, isolated from the rhizosphere of *Leymus chinensis* in saline–alkali soil. This study aims to investigate the role of gamma-aminobutyric acid (GABA)metabolism in regulating the saline–alkali adaptation of strain DJ and its growth-promoting effects on maize under such stress. The results indicated that under saline–alkali conditions, strain DJ upregulated genes associated with GABA metabolism, energizing ion transport and motility. Furthermore, the extracellular GABA concentration in the culture medium of strain DJ rose from 4.04 μmol L^−1^ at 4 h to 11.65 μmol L^−1^ at 12 h, followed by a decline to 7.00 μmol L^−1^ at 24 h, suggesting that GABA potentially promotes cellular growth and physiological activities. Exogenous GABA further enhanced the ion transport capacity, motility, and biomass of strain DJ, confirming the contribution of GABA to improving its saline–alkali adaptability. In maize, strain DJ improved germination (by 7–10%) and seedling growth under saline–alkali conditions, as evidenced by increases in primary radicle length (+2.8 cm), shoot height (+2 cm), and fibrous root number (+4). It upregulated root expression of *GAT1* (threefold), *GABA-T* (1.3-fold), and *SSADH* (1.5-fold), and increased antioxidant enzyme activities (SOD/POD: 1.5-fold; CAT: 1.8-fold). These findings demonstrate that strain DJ enhances the antioxidant capacity of maize through a GABA-mediated pathway and supports energy metabolism for improving tolerance under saline–alkali conditions.

## 1. Introduction

Soil salinization is a global environmental challenge that impairs soil health and diminishes crop yields, thereby significantly threatening food security [1,2]. Saline–alkali soils, characterized by high salt concentrations and elevated pH values, can adversely affect plant growth and even lead to severe physiological stress or plant death. Plant growth-promoting rhizobacteria (PGPR) serve as a crucial factor in regulating ecosystem function and maintaining sustainable agriculture by improving soil nutrient availability and enhancing plant tolerance under abiotic stress conditions [3,4,5,6]. The successful utilization of PGPRs depends on their ability to survive in the soil and adapt to challenging environmental conditions through different mechanisms. Salt-tolerant microorganisms, which can proliferate across a relatively large range of salinities, have evolved two primary strategies to maintain osmotic stability in response to high-salt environments. Specifically, one is the synthesis and intracellular accumulation of compatible solutes (e.g., sugars, polyols, betaines, amino acids, and their derivatives) to balance intra- and extracellular osmotic pressure. The other is the active regulation of ion efflux and input to cope with high-salt environments [7,8,9].

*Enterobacter cloacae* DJ is a plant-beneficial bacterium isolated from saline–alkali environments. Previous studies have revealed its strong adaptability to such conditions, including a chemotactic response toward saline–alkali stimuli. To better understand its adaptive strategies, a comparative proteomic analysis was conducted to investigate the adaptive mechanism of the strain. The relevant experimental procedures and partial findings have been reported in another article [10]. Furthermore, fluorescence quantitative PCR results have verified that genes associated with chemotaxis are involved in adaptation. KEGG and GO analyses revealed the upregulation of proteins involved in butanoate metabolism, flagellar assembly, osmoregulation, and membrane transport systems. Intriguingly, our preliminary data revealed an increase in extracellular gamma-aminobutyric acid (GABA) concentration in the DJ culture under saline–alkali stress. We hypothesized that, intracellularly, a portion of GABA is metabolized through the GABA shunt to enter the tricarboxylic acid (TCA) cycle, providing energy for bacterial motility. In contrast, another portion is secreted into the extracellular environment. When the extracellular GABA reaches a threshold concentration, the GABA concentration in the bacterial culture begins to decrease, and this portion of GABA potentially promotes cellular growth and development. Therefore, this study aims to further investigate the role of GABA in the saline–alkali adaptation of strain DJ in an experimental way.

Furthermore, given that GABA has been well documented to enhance crop tolerance under abiotic stress [11,12,13,14,15], this study also explores how strain DJ improves maize tolerance under saline–alkali stress through GABA-mediated pathways and elucidates the underlying mechanisms.

## 2. Materials and Methods

### 2.1. Chemicals and Reagents

The GABA ELISA kit was obtained from Shanghai Shuangying Biotechnology Co., Ltd. (Shanghai, China). SYBR Green PCR Real Master Mix was provided by Thermo Fisher Scientific (Waltham, MA, USA). The RNA extraction kit was purchased from Tiangen Biotech (Beijing) Co., Ltd. (Beijing, China). The GABA standard and CCCP were supplied by Shanghai Yuanye Bio-Technology Co., Ltd. (Shanghai, China). The assay kits for SOD, POD, and CAT were purchased from Nanjing Jiancheng Bioengineering Institute (Nanjing, China).

### 2.2. Bacterial Strains and Culture Conditions

*Enterobacter cloacae* strain DJ was cultured following a previous protocol [10]. Briefly, cells were grown at 28 °C with shaking (150 rpm) in standard ADF medium. The ADF medium (1 L) was prepared as follows: KH_2_PO_4_ (4 g), Na_2_HPO_4_ (6 g), MgSO_4_·7H_2_O (0.2 g), glucose (2 g), gluconic acid (2 g), citric acid (2 g), and L-Arginine (2 g) or in saline–alkali ADF medium (containing 1.755 g/L NaCl, pH adjusted to 9, the electrical conductivity of the solution was 13.656 dS·m^−1^ at 25 °C). Growth was tracked by measuring OD_600_ on a microplate reader (Flex Station 3, Molecular Devices), and pH changes were monitored using a pH meter during the incubation.

### 2.3. Real-Time Fluorescence qPCR to Validate Proteomics Analysis Results

Total RNA was extracted from DJ bacterial cells cultured for 12 h under test and control conditions using the RNA Purification Kit (Tiangen Biotech Co., Ltd., Beijing, China) according to the manufacturer’s instructions. Reverse transcription was then performed to generate cDNA (Tiangen Biotech Co., Ltd., Beijing, China). Quantitative real-time PCR (qPCR) was carried out using the SYBR Green PCR Real Master Mix (Applied Biosystems, Thermo Fisher Scientific, USA) on an ABI 7500 Real-Time PCR System (Applied Biosystems, USA). The thermal cycling program was as follows: initial denaturation at 95 °C for 5 min; 40 cycles of denaturation at 95 °C for 50 s and annealing at 60 °C for 15 s, followed by extension at 72 °C for 50 s; and a melt curve analysis was subsequently performed to verify amplification specificity. The relative expression levels of target genes were calculated using the 2^−ΔΔCt^ method, with 16S rDNA serving as the internal reference. All primer sequences used for gene detection are listed in Table 1.

### 2.4. Detection of GABA in Bacterial Supernatant

For the determination of GABA concentration in the bacterial culture, the bacterial cells were cultured continuously for 24 h, and culture samples were collected at 4 h intervals for analysis. DJ bacterial cultures were centrifuged at 10,625 *g* to collect the supernatant. GABA concentration was measured with an ELISA kit according to the instructions given by the manufacturer. OD_450_ was recorded with a microplate reader after adding the stop solution, and finally compared with the standard curve of OD versus GABA concentration, which was produced concurrently.

### 2.5. Assessing the Role of GABA in the Growth of DJ Bacteria

DJ bacteria were cultured in saline–alkali ADF medium supplemented with filter-sterilized GABA (20 μmol/L) for the GABA group. A separate group received both filtered GABA and the ABC transporter protein inhibitor CCCP (10 mg/L), designated as the CCCP group. Growth was monitored via OD_600_ measurements over time during the incubation. A bacterial swimming test was also carried out. After cooling, 0.5 μL of the DJ bacterial culture was inoculated into the center of the semi-solid medium (containing 2.8% agar powder) supplemented with GABA and CCCP. After incubation at 28 °C, colony diameters were recorded at 12 and 24 h. Gene expression changes for the ABC transporter protein, bacterial motility proteins, and *KdpB* were analyzed by real-time PCR in different groups, as described previously.

### 2.6. Relationship Between GABA and the Growth-Benefiting Effects of DJ Strain

Maize seeds were soaked in 50 °C water for 1 h, then placed in Petri dishes with 20 in each, and treated with saline–alkali ADF medium and salinity–alkaline ADF-cultured bacterial solution, respectively. After incubation at 25 °C, the germination rates were calculated at 24, 48, and 72 h. The above experimental procedures were repeated three times. The germinated seedlings were cultivated on 1/2 MS medium supplemented with 30 mM NaCl (pH 9) for 15 days, after which fresh weight, primary radicle length, stem height, and adventitious root number were measured. The activities of antioxidant enzymes (SOD, POD, and CAT) in maize seedlings were measured using a commercial kit. Briefly, 15-day-old maize seedlings were frozen in liquid nitrogen and ground into powder. Total soluble proteins were extracted with phosphate buffer (pH 7.4). Protein concentration was determined using a BCA kit, and antioxidant enzyme activities were assayed following the kit instructions, expressed as U/mg prot. Furthermore, total RNA was extracted from the roots of 15-day-old maize seedlings. After reverse transcription to generate cDNA (Tiangen Biotech Co., Ltd., Beijing, China), the expression levels of GABA metabolism-associated genes in maize root samples were quantified by qPCR using the glyceraldehyde 3-phosphate dehydrogenase (*GAPDH*) gene as an internal reference. The thermal cycling program was as follows: initial denaturation at 95 °C for 5 min; 40 cycles of denaturation at 95 °C for 50 s and annealing at 60 °C for 15 s, followed by extension at 72 °C for 50 s; and a melt curve analysis was subsequently performed to verify amplification specificity. The relative expression levels of target genes were calculated using the 2^−ΔΔCt^ method. All primer sequences were listed in Table 1.

### 2.7. Data Analysis

All data were analyzed and graphed using GraphPad Prism 10 and are presented as means ± SDs. Before performing one-way ANOVA, the Shapiro–Wilk test for normality and Levene’s test for homogeneity of variances were conducted, and all data satisfied the assumptions for parametric analysis. One-way ANOVA with Tukey’s post hoc test was used to identify significant differences, with statistical significance set at *p* < 0.05 (indicated by asterisks).

## 3. Results

### 3.1. GO Enrichment and KEGG Pathway Analysis and Validation

**Figure 1:**
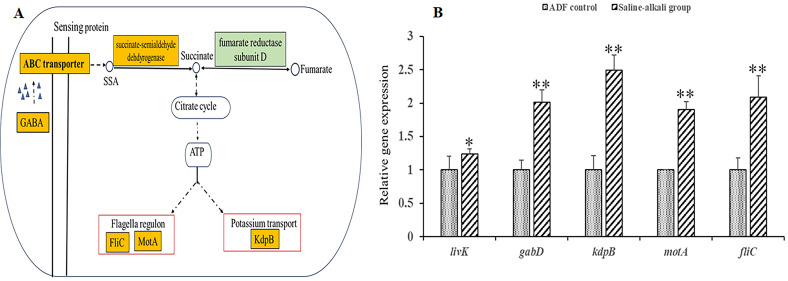
GO enrichment and KEGG pathway analysis and validation. (**A**) Proteins that may be associated with saline–alkali adaptation in DJ bacteria, Yellow: Up-regulated gene expression, Green: Down-regulated gene expression, Solid lines: directly detected pathways, dashed lines: indirectly deduced pathways. (**B**) Validation of gene expression levels in core pathways: compared with the ADF control group, the gene expression level validation results in the saline–alkali group were consistent with the proteomics analysis results. *livK* encodes branched-chain amino acid binding protein, *gabD* encodes succinate-semialdehyde dehydrogenase, *kdpB* encodes potassium-transporting ATPase ATP-binding subunit, *motA* encodes flagellar motor protein MotA, *fliC* encodes flagellar assembly protein, and *frdD* encodes succinate dehydrogenase subunit D. Means ± SDs (*n* = 3 biological replicates, with 3 technical replicates each), * *p* < 0.05, ** *p* < 0.01.

Figure 1A displays the proteins potentially involved in the saline–alkali adaptation of strain DJ, which were identified by proteomic analysis as significantly differentially expressed under saline–alkali conditions compared to the control. In the figure, proteins with upregulated expression are marked in yellow, while those with downregulated expression are marked in green. This study subsequently focuses on analyzing the role of the GABA molecule in the saline–alkali adaptation process of strain DJ.

Quantitative real-time PCR (qPCR) was employed to validate the transcript levels of selected genes associated with core pathways identified through KEGG enrichment analysis, which encode proteins identified in the proteomics analysis, including the two-component system, butanoate metabolism, and bacterial motility. The results demonstrated that key gene expression levels in the core pathways were highly consistent with the proteomics data. Under saline–alkali conditions, expression of genes detected in the experimental groups differed significantly from the control. Specifically, *livK*, *gabD*, *kdpB*, *motA* and *fliC* were upregulated, while *frdD* was downregulated.

### 3.2. GABA Concentration in Supernatant of Strain DJ

**Figure 2:**
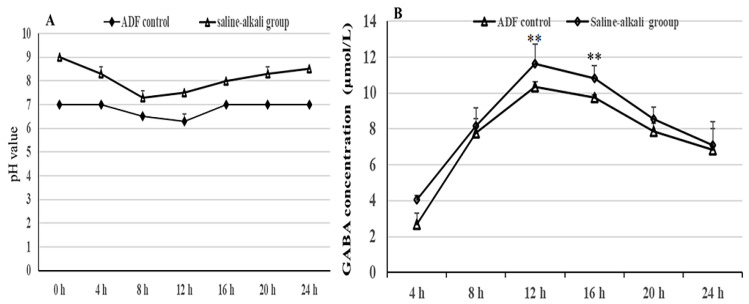
pH changes and GABA concentration in the supernatant of bacteria. (**A**) pH changes during the cultivation period. (**B**) GABA concentration in the supernatant of DJ bacteria: the GABA content in the bacterial culture supernatant was significantly higher in the saline–alkali group than in the control group at 12 and 16 h of cultivation. ** *p* < 0.01 vs. SA group, means ± SDs (*n* = 6).

To clarify the role of GABA in the adaptation of strain DJ to saline–alkali stress, the bacterium was cultured in ADF medium and saline–alkali ADF medium, respectively. The GABA concentration in the bacterial supernatant was measured at different time points using a GABA assay kit. The results indicated that the pH value of the bacterial suspension in the saline–alkali group decreased during the first 8 h of cultivation, with a more pronounced decrease than in the control. The lowest pH value was detected at the 8 h mark, after which it remained relatively low for a period (Figure 2A). Under the same cultivation conditions, the GABA concentration in the bacterial suspension of the saline–alkali treatment group was consistently higher than that of the control group. Measurements at 12 h and 16 h revealed a significant difference between the two groups. The GABA concentration rose from 4.04 μmol L^−1^ at 4 h to 11.65 μmol L^−1^ at 12 h, after which it gradually declined to 7.00 μmol L^−1^ at 24 h, suggesting that this portion of GABA may promote cellular growth and development.

### 3.3. Role of GABA in Growth of Strain DJ

**Figure 3:**
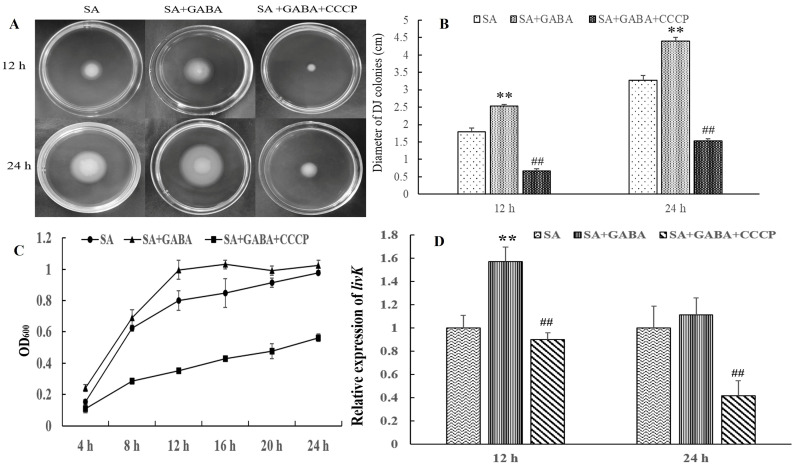
Effects of exogenous GABA on DJ bacteria. (**A**) Semi-solid plate assay, SA: Salinity–alkaline ADF medium, SA + GABA: Exogenous GABA was added to SA medium, SA + GABA + CCCP: GABA and CCCP were added to SA medium. (**B**) The colony diameter: The colony diameters were measured at 12 h and 24 h post-inoculation. The GABA-treated group exhibited significantly larger colony diameters compared to the SA (control) group at both time points, while the CCCP-treated group showed significantly smaller colony diameters than both the control and GABA-treated groups; Means ± SDs (*n* = 3). (**C**) The Optical Density (OD) value of the DJ bacteria: The GABA-treated group exhibited significantly higher OD values compared to the control group, while the CCCP-treated group showed a significantly lower biomass than the control group. Means ± SDs (*n* = 3). (**D**) The relative expression of ABC transporter-encoding genes: In the GABA-treated group, the expression level of the *livK* gene at 12 h was significantly higher than that of the control group, while no significant difference was observed at 24 h. In contrast, the expression of the *livK* gene in the CCCP-treated group was significantly lower compared to the control group. ** *p* < 0.01 vs. SA group, ^##^ *p* < 0.01 vs. SA group, means ± SDs (*n* = 3).

In order to further explore the effect of GABA on the growth of strain DJ under saline–alkali conditions, exogenous GABA (20 μM) and the ABC transporter inhibitor Carbonyl Cyanide 3-Chlorophenylhydrazone (CCCP) (10 μg/L) were employed. Based on proteomic evidence and subsequent qPCR validation indicating the upregulation of the membrane ABC transporter (encoded by *livK*) under saline–alkali stress, we proposed its potential involvement in GABA transport. CCCP, as an inhibitor of the ABC transporter, was expected to restrict the entry of substrate into the cells when added. Due to the addition of exogenous GABA, the optical density (OD) of DJ cultures was sharply higher relative to that of the control group (SA), signifying that exogenous GABA can enhance the biomass of DJ bacteria under saline–alkali conditions. However, co-treatment with GABA and inhibitor CCCP reduced the biomass of DJ bacteria greatly, as shown in Figure 3C. The swim plate assays demonstrated that the colony diameter of DJ bacteria was obviously enlarged when compared to that of the control (SA) after the addition of exogenous GABA, whereas CCCP addition in the culture substantially restricted motility (Figure 3A,B). Furthermore, qPCR analysis of the ABC transporter-encoding gene *livK* under different culture conditions revealed that *livK* expression levels were elevated upon GABA addition and significantly decreased in the presence of CCCP (Figure 3D).

### 3.4. Impact of Exogenous GABA on Expression of Other Critical Genes in Strain DJ

**Figure 4:**
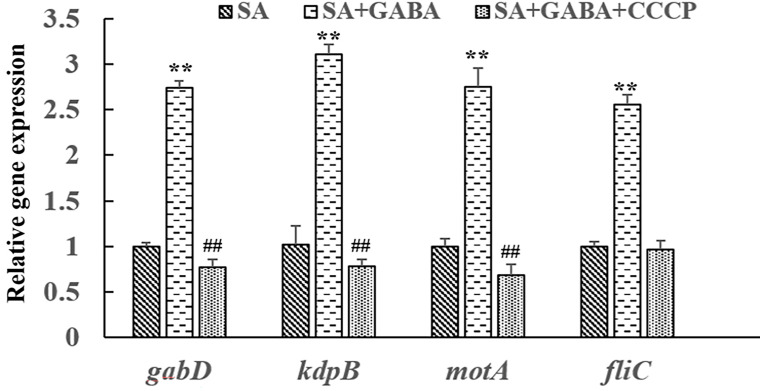
The effect of exogenous GABA on the gene expression levels of DJ bacteria. SA: Salinity-alkaline ADF medium. SA + GABA: Exogenous GABA (20 μM/L) was added to SA medium. SA + GABA + CCCP: GABA(20 μM/L)and CCCP (10 μg/L) were added to SA medium. Means ± SDs (*n* = 3), ** *p* < 0.01 vs. SA group, ^##^ *p* < 0.01 vs. SA group.

Proteomic and qPCR analysis revealed that expression of several critical genes, including the ABC transporter gene (*livK*), the GABA metabolism-associated gene (*gabD*), the potassium ion transporter gene (*kdpB*), and the motility-related genes (*fliC*, *motA*), was substantially upregulated in DJ strain cells following 12 h saline–alkali cultivation. The specific data are presented in Figure 1. To verify whether exogenous GABA affected the expression of the above-mentioned genes, qPCR was employed to analyze the expression of *gabD*, *kdpB*, *motA*, and *fliC*, after GABA treatment. The results are displayed in Figure 4.

As evidenced by the experimental results, the addition of exogenous GABA markedly upregulated the expression of *gabD*, *kdpB*, *motA*, and *fliC* in comparison to the control, with obvious differences observed. In contrast, when the inhibitor CCCP was added, the expression of *gabD*, *kdpB*, and *motA* decreased relative to the control. Experimental results indicated that exogenous GABA elevated the expression of genes involved in GABA metabolism and bacterial motility in DJ bacterial cells. GABA is metabolized within the cells, potentially providing energy support for ion transport and motility, or influencing these processes through other mechanisms. Together, these findings suggest that GABA may play a key role in facilitating the adaptation of DJ bacteria to saline–alkali environments.

### 3.5. Influence of the DJ Bacterial Strain on the Growth and Development of Maize (Zea mays) Under Saline–Alkali Conditions

**Figure 5:**
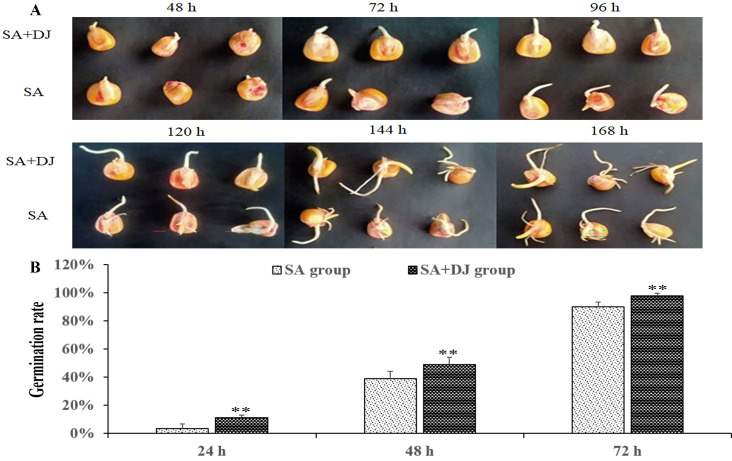
Impact of the DJ strain on maize seed germination rate under saline–alkali conditions. SA: Maize materials treated with salinity–alkaline ADF medium served as the control group; SA + DJ: Maize materials treated with DJ bacterial culture (cultured in SA medium for 12 h) served as the experimental group. (**A**) Representative images of seed germination. (**B**) Germination rate statistics within 72 h. Means ± SDs (6 biological replicates, with 15 maize seeds per treatment each time) ** *p* < 0.01 vs. SA group.

Under saline–alkali conditions, the GABA concentration in the supernatant of the DJ strain was elevated. In this study, we hypothesize that this secreted GABA may contribute to the plant growth-benefiting effects of the DJ strain. To validate the potential of this hypothesis, maize seeds were treated with the DJ strain cultured in saline–alkali ADF medium (Bacterial OD_600_ was approximately 0.6–0.8), and germination rates were recorded at 24, 48, and 72 h. The results revealed that, under saline–alkali conditions, the DJ strain significantly enhanced maize seed germination rates by 7–10% compared to the control groups, as depicted in Figure 5A,B.

**Figure 6:**
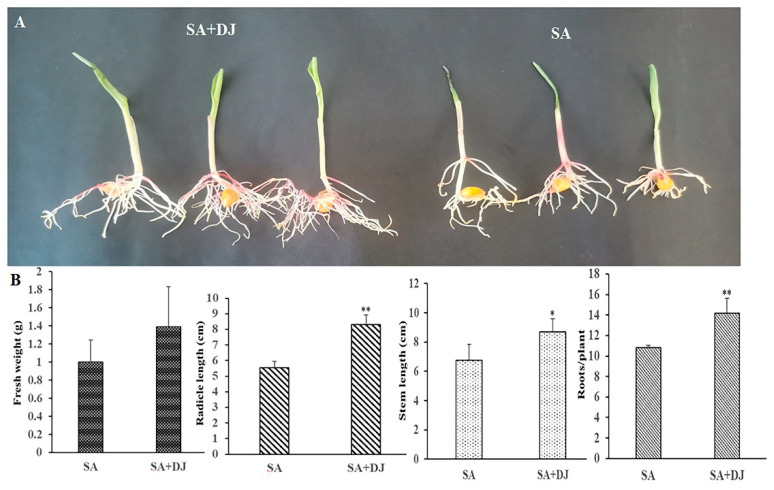
Influence of the DJ bacterial strain on the growth and development of maize under saline–alkali conditions. SA: Maize materials treated with salinity–alkaline ADF medium served as the control group; SA + DJ: Maize materials treated with DJ bacterial culture (cultured in SA medium for 12 h) served as the experimental group. (**A**) Representative sseedlings of control group and DJ-treated group. (**B**) Statistical comparison of growth indices, including fresh weight, stem height, primary radicle length, and adventitious root number. Means ± SDs (6 biological replicates, with 15 maize seedlings per experiment), * *p* < 0.05, ** *p* < 0.01 vs. SA group.

Subsequently, maize seeds treated with DJ bacterium were germinated and grown on 1/2 MS medium supplemented with 30 mM NaCl (pH 9) for 15 days, after which seedling fresh weight, stem height, primary radicle length, and adventitious root number were determined. As illustrated in Figure 6A,B, following DJ strain treatment, maize seedlings grown under saline–alkali conditions exhibited increased fresh weight, primary radicle length (+2.8 cm), stem height (+2 cm), and adventitious root number (+4) compared to the control, with extremely significant differences observed in primary radicle length, stem height and adventitious root number.

**Figure 7:**
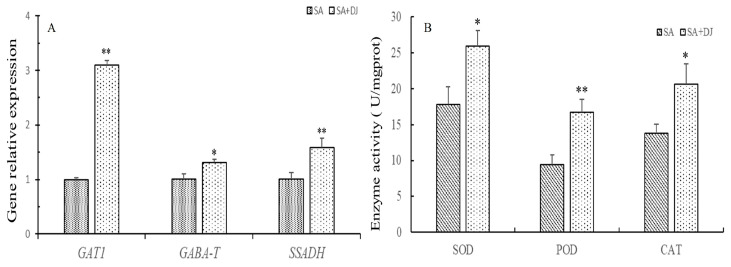
Effects of DJ bacteria on the expression of related genes and antioxidant enzymes in maize seedlings under saline–alkali conditions. (**A**) Effects of DJ bacteria on the expression of GABA transport and metabolism-related genes in maize seedlings. (**B**) Effects of DJ bacteria on antioxidant enzymes in maize seedlings. SA: Maize materials treated with salinity–alkaline ADF medium served as the control group, SA + DJ: Maize materials treated with DJ bacterial culture (cultured in SA medium for 12 h) served as the experimental group. Means ± SDs (*n* = 3) * *p* < 0.05, ** *p* < 0.01 vs. SA group.

To examine whether the growth-promoting effect of the DJ strain was associated with GABA present in the bacterial culture, expression of GABA receptor genes and GABA metabolism-related genes in maize roots was analyzed. The specific examination results are presented in Figure 7A,B. Quantitative fluorescence analysis showed that strain DJ significantly upregulated the expression of *GAT1* (a GABA receptor gene) in maize seedling roots, reaching three times that of the control. Similarly, qPCR analysis revealed that the expression levels of GABA shunt key genes, *GABA-T* and *SSADH*, were increased by 1.3- and 1.5-fold, respectively, following DJ treatment (Figure 7A). Meanwhile, antioxidant enzyme activities (SOD, POD, and CAT) in maize seedling leaves were also enhanced under saline–alkali conditions with DJ treatment. Specifically, SOD and POD activities were 1.5-fold higher, and CAT activity was 1.8-fold higher than the control (Figure 7B). Collectively, these results indicate that GABA from the DJ bacterial solution is taken up by maize root cells via receptor proteins, enters the GABA shunt to provide energy under stress, and enhances antioxidant enzyme activity, thereby likely improving plant resistance and promoting growth under saline–alkali conditions.

## 4. Discussion

The preliminary research results indicated that the DJ bacteria possess a degree of adaptability to saline–alkali conditions. To explore the molecular basis of this adaptation, a proteomic analysis was conducted, and the detailed methodology and results were described in previously published articles [10]. Core pathway analyses using GO and KEGG revealed that proteins with differential expression were primarily enriched in pathways involved in bacterial chemotaxis, two-component systems, butanoate metabolism, quorum sensing, and flagellar assembly. Genes associated with bacterial chemotaxis were analyzed previously. The core pathway analysis further showed that in the cells of DJ bacteria, GABA is converted into succinic semialdehyde under the catalysis of 4-aminobutyrate aminotransferase. Under saline–alkali stress conditions, proteomic data indicated upregulation of succinic semialdehyde dehydrogenase, which catalyzed the subsequent conversion of succinic semialdehyde into succinate. Next, this succinate could enter two possible metabolic pathways. One route involved conversion of succinate into fumarate by the action of fumarate reductase; however, proteomic data suggested that expression of the D subunit of fumarate reductase was downregulated in this pathway. Therefore, the succinate derived from GABA metabolism was more likely to enter the citric acid cycle. Through this cycle, energy could be released, providing essential energy support for DJ survival in saline–alkali environments and contributing to cellular metabolic regulation. Quantitative real-time PCR (qPCR) validation of the expression levels of *livK*, *gabD*, *kdpB*, *motA*, *fliC*, and *frdD* yielded results that were consistent with the proteomic data, confirming that the products of these genes played a critical regulatory role in facilitating the adaptation of the DJ bacterium to saline–alkali conditions.

Saline–alkali stress induces cellular dehydration, prompting bacteria to employ diverse adaptive strategies. The K^+^ uptake system serves as a rapid response mechanism that quickly alleviates osmotic stress, while bacteria also accumulate compatible solutes to maintain long-term osmotic balance [16,17,18,19]. In strain DJ, the expression of kdpB, a gene involved in K^+^ transport, was upregulated under saline–alkali conditions (Figure 1), which may contribute to its immediate response to hyperosmotic stress. Concurrently, elevated GABA levels were detected in the supernatant of DJ bacterial cultures under the same stress, as given in Figure 2. This stress-induced GABA may also participate in osmoregulation.

In another study, based on some experiments, we preliminarily confirmed that the Na^+^ concentration in saline–alkali environments is associated with the motility of the DJ strain. Specifically, elevated Na^+^ concentrations enhanced the motility of the DJ strain, a process requiring substantial energy support [20]. Correspondingly, expression of *motA* and *fliC* was upregulated in DJ bacterial cells under saline–alkali conditions. It is known that *motA* encodes the flagellar motor protein MotA, and *fliC* encodes the flagellar assembly protein FliC. These two proteins are associated with the motility of the DJ bacteria, and GABA metabolism may provide the energy required for the movement of the DJ bacteria. Under saline–alkali conditions, the GABA concentration in the DJ bacterial broth was significantly higher than that in the control group at 12 and 16 h of cultivation, peaking at 12 h and subsequently decreasing. Based on these findings, we hypothesize that under saline–alkali stress, DJ bacteria synthesize more GABA. Some of this GABA might be catabolized intracellularly, potentially contributing to bacterial motility and other physiological activities, while some may be secreted extracellularly. Following the gradual accumulation of extracellular GABA to a critical concentration, a subsequent decrease in its level in the culture medium was observed, which implies that GABA could play a regulatory role in subsequent cellular processes. Furthermore, we employed exogenous GABA and the ABC transporter inhibitor to examine the influence of GABA on the DJ strain. As a result, we found that the addition of GABA to the liquid medium significantly increased the biomass of the DJ strain compared to the control. In a semi-solid medium, GABA supplementation also markedly expanded the migration radius of the DJ strain under saline–alkali conditions, as demonstrated in Figure 3. Moreover, qPCR analysis confirmed that, under stress, expression of *livK*, *gabD*, *kdpB*, *motA,* and *fliC* in the DJ strain cultured with added GABA was much higher in comparison to that in the control. In contrast, adding CCCP inhibitor resulted in significantly lower intracellular expression of *livK*, *gabD*, *kdp*B, motA, and fliC. The experimental findings indicated that exogenous GABA could enhance both the motility and biomass of the DJ strain under saline–alkali environment, thereby improving its adaptability to such stress. The stress-induced elevation of GABA concentration observed in the DJ bacterial culture may be linked to both osmotic regulation and energy metabolism of the strain. After 12 h of cultivation, the GABA concentration gradually decreased, suggesting that GABA in the bacterial culture medium may exert effects similar to those of exogenous GABA.

Numerous studies have established that GABA can function as both a defense metabolite and a signaling molecule in various physiological processes, assisting plants in responding to biotic and abiotic stresses [11,21,22,23]. It is documented in multiple studies that plant roots can take up exogenous GABA, which enhances plant resistance under various abiotic stress conditions [11,15,24,25,26]. Based on our experimental results, it is evident that the GABA concentration increased in the DJ strain culture under saline–alkali conditions. We therefore hypothesized that this bacterially derived GABA acts as an exogenous source, enhancing the germination rate of maize seeds and promoting the growth of maize seedlings under such stress. Treating maize seeds with DJ bacterial culture incubated for 12 h increased the germination rate by approximately 10% under saline–alkali conditions. Moreover, this treatment significantly enhanced the primary radicle length and adventitious root number of maize seedlings under saline–alkali conditions, thereby aiding nutrient absorption from the environment during the early growth period; however, no significant effect on seedling fresh weight was detected, which may be attributed to the incomplete leaf expansion and consequently low organic matter accumulation at this stage.

Using quantitative fluorescence analysis, we further detected expression of the GABA receptor gene *GAT1* and its metabolism-related genes in maize roots. In plant cells, GABA is primarily metabolized via the mitochondrial GABA shunt pathway. In this pathway, GABA is first converted into succinic semialdehyde (SSA) by GABA transaminase (GABA-T). Subsequently, SSA is oxidized by NAD^+^-dependent succinic semialdehyde dehydrogenase (SSADH) and ultimately transformed into succinate, which then enters the tricarboxylic acid (TCA) cycle [27,28]. The qPCR results confirmed that after treatment with DJ bacterial solution, expression of the GABA receptor gene *GAT1* was upregulated in maize seedling roots (3-fold). Simultaneously, expression of the *GABA-T* and *SSADH*, key genes involved in the GABA shunt pathway, was also elevated markedly, with great differences observed relative to the control group (Figure 7A). Antioxidant enzymes serve as a “biochemical shield” in plants under stress, scavenging ROS and maintaining cellular redox homeostasis [29,30,31]. Our study unveiled that the activities of SOD, CAT, and POD were significantly increased in maize seedlings treated with DJ bacteria than in the control group (SOD/POD: 1.5-fold; CAT: 1.8-fold) (Figure 7B). These findings are consistent with previous reports that exogenous GABA can bolster plant resistance under abiotic stress [32,33].

## 5. Conclusions

The present study suggests a potential role of GABA-mediated pathways in strain DJ-induced salinity tolerance; however, further validation through functional genomics approaches is required to conclusively substantiate these observations. In summary, the findings of this study indicate that *Enterobacter cloacae* strain DJ may adapt to saline–alkali environments by modulating genes involved in GABA metabolism-related pathways. Some of the synthesized GABA appears to be associated with intracellular catabolism and entry into the TCA cycle, which may correlate with or contribute to energy provision for essential activities of the DJ strain, including ion transport and motility related to niche alteration. Some GABA is secreted extracellularly and could play a regulatory role in subsequent cellular processes. Treatment with the DJ strain increased the germination rate of maize seeds under saline–alkali conditions. The GABA in the bacterial culture was taken up by plant cells via transport proteins and, through the TCA shunt pathway, supplied energy that promoted seedling growth, thereby increasing the fresh weight, stem length, and adventitious root number in maize seedlings. This process also boosted antioxidant enzyme activity, thus strengthening the resistance of maize to saline–alkali stress.

Although this study has investigated the saline–alkali adaptability and growth-promoting mechanisms of strain DJ through experimental design, certain limitations remain. Future studies should incorporate functional genomics validation, including the use of overexpression or CRISPR interference techniques, to functionally characterize key genes involved in GABA metabolic pathways (e.g., glutamate decarboxylase-encoding genes and GABA transporter genes), thereby elucidating their direct roles in saline–alkali adaptation.

## Figures and Tables

**Table 1:** Primer sequences for quantitative PCR assay.

Primer Name for Bacterial qPCR	Primer Sequences (5′–3′)
16S-F	GCCGCAGCATCGTTATTCT
16S-R	TCTCGCCGTAACGCATAT
livK-F	CGCAATATACCAACGGCATCT
livK-R	CACGGAGGCATAGGAGTAGAG
gabD-F	CGCAATATACCAACGGCATCT
gabD-R	TCAGGAAGGCATCGGCAAT
kdpB-F	GCCGCAGCATCGTTATTCT
kdpB-R	TCTCAACCAGATTGTCCACTTC
motA-F	GGCACCTTGGAGCACTCTAT
motA-R	CGACGGAACAGCAATGGAATA
fliC-F	GTAACGCCAACGACGGTATC
fliC-R	CGGTAGCAGCCTGAACAGA
frdD-F	TGATTGTCCTGCCGCTGTG
frdD-R	GATGGTTGCCAGACCGTAGAA
Primer name for plant qPCR	Primer sequences (5′–3′)
GAPDH-F	CAGCCAAGGATTGGAGAGGT
GAPDH-R	ACCACGGACACATCAACAGT
GAT1-F	AAGCGGCTGAAGCAGATGG
GAT1-R	AAGGTGACGAGGTCGGAGA
GABA-T-F	CGCTGGTGGTGTTATCCTCC
GABA-T-R	CAGTAATGACCTCATCCGCAAT
SSADH-F	GACGCATACGATGGCAAGAC
SSADH-R	TACTGTGAGCAGAAGCAATAGC

## Data Availability

The datasets used and/or analyzed during the current study are available from the corresponding author on reasonable request.

## Abbreviations

The following abbreviations are used in this manuscript:

PGPR	Plant growth-promoting Rhizobacteria
KEGG	Kyoto Encyclopedia of Genes
GO	Gene Ontology
GABA	Gamma aminobutyric acid
TCA	Tricarboxylic acid
qPCR	Quantitative real-time PCR
CCCP	Carbonyl Cyanide 3-Chlorophenylhydrazone
OD	Optical density
SSA	Succinic semialdehyde
GABA-T	GABA transaminase
SSADH	Succinic semialdehyde dehydrogenase
GAPDH	Glyceraldehyde 3-phosphate dehydrogenase
SOD	Superoxide dismutase
POD	Peroxidase
CAT	Catalase
ADF	DF (Dworkin and Faster) medium containing 2 g arginine
SYBR	Synergy brands
MS	Murashige–Skoog
